# Influence of trait empathy on the emotion evoked by sad music and on the preference for it

**DOI:** 10.3389/fpsyg.2015.01541

**Published:** 2015-10-27

**Authors:** Ai Kawakami, Kenji Katahira

**Affiliations:** ^1^Brain Science Institute, Tamagawa UniversityTokyo, Japan; ^2^School of Science and Technology, Kwansei Gakuin UniversityHyogo, Japan

**Keywords:** trait empathy, perspective taking, fantasy, sad music, emotional response, liking sad music, 12-year-old children

## Abstract

Some people experience pleasant emotion when listening to sad music. Therefore, they can enjoy listening to it. In the current study, we aimed to investigate such apparently paradoxical emotional mechanisms and focused on the influence of individuals’ trait empathy, which has been reported to associate with emotional responses to sad music and a preference for it. Eighty-four elementary school children (42 males and 42 females, mean age 11.9 years) listened to two kinds of sad music and rated their emotional state and liking toward them. In addition, trait empathy was assessed using the Interpersonal Reactivity Index scale, which comprises four sub-components: Empathic Concern, Personal Distress, Perspective Taking, and Fantasy (FS). We conducted a path analysis and tested our proposed model that hypothesized that trait empathy and its sub-components would affect the preference for sad music directly or indirectly, mediated by the emotional response to the sad music. Our findings indicated that FS, a sub-component of trait empathy, was directly associated with liking sad music. Additionally, perspective taking ability, another sub-component of trait empathy, was correlated with the emotional response to sad music. Furthermore, the experience of pleasant emotions contributed to liking sad music.

## Introduction

Some people like listening to sad music, while others dislike it. Why do sad-music lovers prefer to listen to sad music? Given that sadness is an unpleasant emotion (Russell, 1980), it seems paradoxical that some people want to listen to sad music in the first place. This mystery has been an important issue in esthetics since ancient times (Aristotle, 1986), and it continues to attract attention (Kivy, 1989; Robinson, 1994; Davies, 1997; Levinson, 1997).

We have been trying to clarify this paradoxical phenomenon with an empirical approach, through a set of experiments (Kawakami et al., 2013a,b). In these studies, we differentiated emotion into *perceived* and *felt* aspects, which enabled us to explain the paradox noted above. A perceived emotion is that which we hear being expressed in a musical piece, and a felt emotion is that which is actually induced by a musical piece. Gabrielsson (2002) defined congruent and incongruent patterns of relationships between perceived and felt emotions as having positive and negative relationships, respectively. Thus, based on Gabrielsson’s (2002) notion of a negative relationship, it is possible to explain the paradox of why people listen to music that evokes sadness in listeners. As Kawakami et al. (2013b) suggested, people might want to listen to music that was perceived to express sadness because, in addition to sadness, that music evoked pleasant emotions in them.

In addition, empirical evidence regarding the various kinds of emotions experienced in response to sad music (felt emotion) has been reported recently. Vuoskoski et al. (2012) examined emotional responses while participants listened to musical excerpts (felt emotion), and found that sad music induced not only sadness but also positive emotions such as nostalgia and peacefulness. Taruffi and Koelsch (2014) conducted an online survey and obtained similar data. Moreover, Kawakami et al. (2013b) provided empirical data demonstrating the difference between perceived and felt emotions for identical sad-music excerpts. While the participants perceived the musical excerpts as highly sad, they reported that they did not feed the deep sadness, and also experienced other types of emotions, such as romantic emotion. These studies have revealed the common finding that though sad music actually does induce sadness, pleasant emotions can be also induced simultaneously.

If this is true, *why* do we feel such ambivalent emotions while listening to sad music? Kawakami et al. (2013b, 2014) attempted to explain the underlying mechanism using the term “vicarious emotion.” They focused on the nature of negative emotional stimuli in an esthetic context, where they did not pose any real threat for survival. According to Kawakami et al. (2013b, 2014), the opportunity for positive emotions to be evoked occurs due to the vicarious nature of the emotions induced by sad music. Besides this theoretical consideration, some empirical studies focusing on individual differences in emotional responses to sad music have revealed an association between individual traits and liking sad music (Garrido and Schubert, 2011; Vuoskoski et al., 2012). These studies addressed the trait of empathy, an emotional response that occurs vicariously.

### Empathy and Esthetic Experience

The term “empathy” is currently used exclusively in interpersonal contexts, and researchers in different disciplines define empathy from a broad range of individual perspectives (Wispé, 1987). Among the many current scholarly definitions of empathy, a casual definition that may be familiar is “sharing the perceived emotion of another” (Eisenberg and Strayer, 1987, p. 5). This notion of empathy was first introduced by Lipps (1903, 1905), and is typically considered the origin of this term (Wispé, 1987). However, it is important to note that Lipps did not initially include the interpersonal context in his use of the term. In fact, the concept that he adopted was “einfühlung,” which evolved from German esthetics, and was used to discuss esthetic cognition. It is therefore surprising that, in contrast, the modern usage of the concept of empathy is nearly always reserved for interpersonal contexts.

First, Lipps applied the idea of einfühlung to the esthetics of visual form (Currie, 2011). For Lipps, “einfühlung” meant that an observer projects himself or herself into a perceived object as the only way to appreciate certain esthetic aspects (Wispé, 1987). Later, Lipps (1905) extended his usage of “einfühlung” beyond esthetic appreciation and used it to explain how a person understands the consciousness of others. Thus, the target of einfühlung was expanded beyond esthetic objects, to people (Chismar, 1988). Titchener (1909) borrowed Lipps’ notion of einfühlung, and translated it as “empathy” via the Greek word “empatheia,” which means “in suffering” or “in passion” (en + pathos). This was the first usage of the term “empathy.”

Considering the way in which the term “empathy” emerged, we cannot avoid predicting that empathic ability would be associated with the esthetic emotional experience. Therefore, we are convinced that this suggests that emotion in art, not only in an interpersonal context, should be examined from the perspective of empathic ability.

### Trait Empathy, Emotional Responses, and Liking Sad Music

In fact, some recent studies have suggested that individual differences in dispositional empathy are associated with one’s preference for sad music (Garrido and Schubert, 2011; Vuoskoski et al., 2012). These studies have investigated the effect of trait empathy on liking sad music using the Interpersonal Reactivity Index (IRI; Davis, 1980, 1983). Among the four sub-scales of the IRI [Empathic Concern (EC), Personal Distress, Perspective Taking, and Fantasy (FS)], EC and Fantasy (FS) were correlated with liking sad music (Garrido and Schubert, 2011; Vuoskoski et al., 2012). Outside the field of music, people with high trait empathy have shown a stronger emotional response to (and greater enjoyment of) a sad movie than did people with low trait empathy (De Wied et al., 1994). Based on these findings, a focus on trait empathy might provide important clues to shed light on the mechanism underlying the ambivalent emotions that occur in response to sad music.

Besides the finding that trait empathy is associated with liking sad music, trait empathy such as FS has been found to affect the intensity of emotional responses to sad music, which in turn is an important predictor of music preference (Vuoskoski et al., 2012). In addition, regardless of the kind of music, the degree of preference for specific music is affected by positive emotional reactions and is suppressed by negative reactions (Ladinig and Schellenberg, 2012).

**FIGURE 1:**
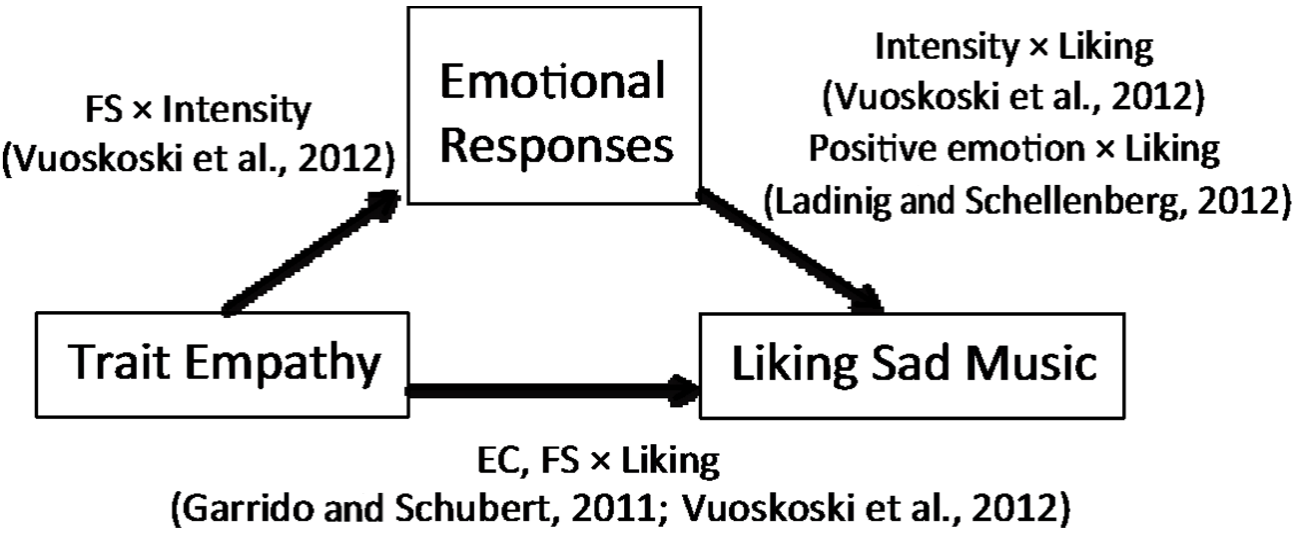
**Relationships between trait empathy, emotional response to sad music, and liking sad music**.

Given the findings described above, in the present study, we proposed a model that explains the relationships between trait empathy, emotional responses to sad music, and preference for the same (**Figure 1**). Previous studies have revealed pairwise relationships between these three variables, including the relationship between FS and intensity of one’s emotional response to sad music (Vuoskoski et al., 2012). However, the specific association between the aspects of trait empathy and an ambivalent emotional response to sad music has not yet been addressed so far.

The associations between the sub-components of trait empathy (EC, FS) and liking sad music as well as the contribution of positive emotions to music preferences have been demonstrated in earlier studies. Therefore, it would be assumed that the positive emotional response to sad music is also associated with these sub-components. However, preferences might be explained not only indirectly through positive emotions, but also directly by trait empathy. Thus, it would be inappropriate to regard emotional response to and preference for music as identical. Hence, it is necessary to investigate the effect of trait empathy on emotional response to sad music within the comprehensive model illustrated in **Figure 1**.

Therefore, in the present study, we conducted a music-listening experiment to examine the relationships among the study variables (trait empathy, emotional response to sad music, and liking sad music). We used the IRI scale to measure individuals’ trait empathy. The IRI has been used in previous studies that examined the relationship between responses to sad music and empathy (Vuoskoski and Eerola, 2012; Vuoskoski et al., 2012; Taruffi and Koelsch, 2014). Therefore, this scale is useful to compare the results of the present study with those of previous ones. In addition, the IRI is a multi-dimensional measure of empathy that measures the emotional components: EC and PD, and cognitive components: PT and FS. According to Davis (1980), these sub-components of the IRI measure different aspects of the process in which empathic response arises. Therefore, findings obtained by using the IRI would be useful for future studies to identify the components of empathy that are related to the pleasant emotion induced by sad music.

The subjects in the present study included children in elementary school because this age group represents the developmental stage during which empathy emerges. For example, Litvack-Miller et al. (1997) measured dispositional empathy in second, fourth, and sixth grade students by using the IRI. They demonstrated that the score on the EC sub-scale of the IRI increased with age. We predicted that employing this age group would highlight individual differences in empathy and allow us to examine the possible relationship between trait empathy and responses to sad music.

As a whole, based on the findings from previous studies, this study aimed to test the following three hypotheses: (1) The sub-components of trait empathy, notably EC and FS, would explain the degree of preference for sad music directly or indirectly, mediated by the emotional response. (2) FS would facilitate an emotional response to sad music. (3) An emotional response to music, particularly a pleasant emotional response, would contribute to liking sad music.

## Materials and Methods

### Participants

Participants included 42 female and 42 male sixth-grade students from an elementary school in Japan. The mean age of the participants was 11.9 years (*SD* = 0.33).

### Materials

We used the following two musical pieces: (1) Granados’s *Allegro de Concierto (G minor)*, played at quarter note = 70 and (2) Glinka’s *La Separation (F minor)*, played at quarter note = 80. These pieces were also used in a former study in which the relationship between emotion perceived in a certain music and those induced by that music was examined (please refer to the musical scores used in Kawakami et al., 2013b).

Kawakami et al. (2013b), selected little-known pieces as musical stimuli because individuals often associate a particular memory or event with well-known music. If the participants knew the music used in our experiment, some listeners might have an emotional response related to that particular memory or event. We aimed to investigate the emotional experience induced purely by music. Therefore, we used two pieces that were reported as unfamiliar by all the participants in the previous study (Kawakami et al., 2013b).

These two pieces were found to be perceived as sad music in the previous study (Kawakami et al., 2013b). Regarding the children’s ability to perceive emotions expressed by music, Gregory et al. (1996) reported that children aged 7–8 years could relate major and minor modes to happy and sad, respectively. Furthermore, Kastner and Crowder (1990) demonstrated that even children aged 3 years are capable of such an association. Given these findings, we believed that the two pieces (both in minor mode) utilized in the present study would also be perceived as sad music by the participants.

The sound level ranged from 58.4 dB (C) to 72.5 dB (C) for Granados’s Allegro de Concierto (G minor), and from 46.5 dB (C) to 70.5 dB (C) for Glinka’s La Separation (F minor). Both pieces were just under 30 s long.

### Self-report Measures of Emotional State

We asked the participants to report their experienced emotions after listening to the pieces of music, which they rated using 50 emotion-related descriptive words and phrases on a scale that ranged from 1 (not at all) to 5 (very much).

These 50 descriptive words and phrases were based on the 62 items used in a previous study (Kawakami et al., 2013b). The participants included in Kawakami et al. (2013b)’s study were adults; therefore, for the current study, we selected only those words that were appropriate for children. Additionally, we obtained a list of 69 adjectives (words and phrases) intelligible to elementary school children from a music teacher. This list has been developed by a society for research on music appreciation and is repeatedly used in music appreciation classes by music teachers. While the words and phrases on the list were chosen to describe the properties of a music piece, most of items seemed to be applicable to the listener’s emotional state. Taken together, 50 items were selected for children to rate their emotional states. Further, as done in previous studies (Garrido and Schubert, 2011; Ladinig and Schellenberg, 2012; Vuoskoski and Eerola, 2012; Vuoskoski et al., 2012), we also asked the participants to rate how much they liked the sad music using a scale that ranged from 1 (not at all) to 5 (very much). We consulted the music teacher and a coordinator at the elementary school when creating the questionnaire and selecting the adjectives.

### Interpersonal Reactivity Index (IRI)

After the participants described their emotional state, they responded to the IRI on a scale ranging from 1 (not at all) to 5 (very much). We used the IRI for children developed by Hasegawa et al. (2009). They translated the scale by Davis (1980) into Japanese, and modified it for use with children. Its reported reliability is 0.79 for PT, 0.69 for EC, 0.80 for PD, and 0.79 for FS (Cronbach’s alpha). The IRI for children includes 30 items (EC: seven items; PT: nine items; PD: seven items; and FS: seven items). Since Hasegawa et al. (2009) recommended this scale for students in the fourth-grade or higher, the scale was considered appropriate for the sixth-grade students who participated in our experiment.

### Procedure

The present study was approved by Tamagawa University Institutional Review Board in terms of the ethics and safety of psychological experiments and brain activity measurement. The study was conducted in conformity with the ethical standards provided by the review board. We did not obtain informed written consent from parents of students because the music-listening experiment was conducted during a regular music class. Given the time limitation of the class, and individual differences of the understanding about the procedure of informed consent expected in this age group, it was assumed that it would be difficult to obtain informed written consent from all the children. Therefore, on the advice of members of a local board of education, we obtained oral consent from the principal and the music teacher of the students. Furthermore, the experiment was conducted under the supervision of members of a local board of education. In the music-listening experiments, the participants performed two tasks. The first was to report their emotional state after listening to each piece of music. The participants listened to two pieces, both in a minor mode. Before starting the first task, they practiced reporting their emotional state twice. In the practice trials, we used two very short pieces of music (about 15 s long) that were different from those used in the main task.

Based on the music teacher’s advice, we took care not to distribute the questionnaire listing descriptive emotional words and phrases before playing the music so that the participants could focus on the music. If we had distributed the questionnaire in advance, some children might have attended to the questionnaire rather than to the music or started reporting their emotional state before the music ended. After all the participants had completed the questionnaire, we collected them and then played the second piece.

The second task was to complete the IRI. Before both tasks, we explained that these tasks were not associated with their academic results and that there was no correct or wrong answer. We asked participants to respond to the musical emotion scale and the IRI honestly. We did not ask participants to provide their name.

### Statistical Analysis

First, the relationship between trait empathy and liking sad music was examined. In addition, the correlations between the IRI sub-components, and partial correlations with liking sad music were investigated for each sub-component.

Second, a factor analysis was performed on the emotion scale data to determine the possible factors underlying the emotional response to the musical stimuli using 168 data points: 2 (Glinka’s *La Separation in F minor* and Granados’s *Allegro de Concierto in G minor*) × 84 participants.

The relationships between the identified factors and other variables were then examined. All statistical analyses were conducted using the IBM SPSS Statistics 22 software. Finally, a path analysis was conducted to evaluate the model proposed in this study and to test the hypothesis that the sub-components of trait empathy influence preference for sad music through one’s emotional response. The path analysis was performed in the IBM SPSS Amos 22 software, using maximum likelihood estimation. The model solutions were assessed using a combination of the following fit indices: chi-square, adjusted goodness of fit index (AGFI), comparative fit index (CFI), and root mean square error of approximation (RMSEA).

## Results

### Correlation between Trait Empathy and Liking Sad Music

Correlation analyses were conducted to investigate the association of trait empathy with liking sad music. The scores of individuals’ global empathy were calculated by averaging the ratings of all items on the IRI for children. The scores for the sub-components of trait empathy were calculated as averaged ratings of the items representing each sub-component. Regarding the preference variable, the degree to which individuals like sad music was assessed by averaging the ratings for the two pieces of music.

The Pearson’s correlation coefficients between variables have been shown in **Table 1**. The correlation analyses revealed that three sub-components of trait empathy (EC, PT, and FS) were significantly correlated with liking sad music (*r* = 0.28, *p* < 0.05 for EC; *r* = 0.35, *p* < 0.01 for PT; and *r* = 0.27, *p* < 0.05 for FS).

**Table 1:** Correlation coefficients between variables.

	Liking	EC	PD	PT	FS	Global
Liking	-					
EC	0.28^∗^	-				
PD	-0.005	0.16	-			
PT	0.35^∗∗^	0.76^∗∗^	0.05	-		
FS	0.27^∗^	0.47^∗∗^	0.29^∗∗^	0.68^∗∗^	-	
Global	-0.004	-	-	-	-	-

*^∗^*p* < 0.05, ^∗∗^*p* < 0.01.*

In line with an earlier study (Vuoskoski et al., 2012) that suggested that EC and FS, in addition to global empathy, correlated significantly with liking sad music, our data also showed a significant relationship between both EC and FS, and liking sad music. Moreover, in the current study, PT was also correlated with liking sad music.

While EC did not correlate with PD (*r* = 0.16, *p* = 0.14), and PT did not correlate with PD (*r* = 0.05, *p* = 0.66), there were significant correlations between some of the sub-components of trait empathy. Specifically, EC correlated with both PT (*r* = 0.76, *p* < 0.01), and FS (*r* = 0.47, *p* < 0.01), while PD correlated with FS (*r* = 0.29, *p* < 0.01), and PT correlated with FS (*r* = 0.68, *p* < 0.01). Therefore, partial correlation analyses were performed to assess the associations between each sub-component and preference for sad music while controlling for the rest of the sub-components. No other significant associations were found between the sub-components of trait empathy and liking sad music (EC: *r* = 0.04, PD: *r* = -0.05, PT: *r* = 0.13, FS: *r* = 0.07). Consequently, we decided to conduct path analyses to examine the relationship between trait empathy and liking sad music as mediated by the emotional response to music. Regarding the emotional response to music, we conducted a factor analysis, as shown in the following section.

### Emotional Response to Music

A factor analysis was performed to identify the underlying structure of the 50 items on the musical emotion scale. The result of the factor analysis, using the principal factor method with promax rotation on the responses of the 84 participants for the two musical stimuli (*n* = 168), revealed a three-factor solution. We conducted a reliability test and deleted seven items so as to maximize the Cronbach’s α. These factors explained 53.04% of the total variance.

The 43 emotion-related descriptive words and phrases with factor loadings have been reported in **Table 2**. Twenty-seven emotion-related descriptive words and phrases, such as “tender,” “fascinated,” and “peaceful,” were included in Factor 1 (Cronbach’s α = 0.971), “sweet emotion.” Ten other words or phrases, such as “sad,” “lonely,” and “gloomy,” were included in Factor 2 (Cronbach’s α = 0.920), “tragic emotion.” In the third factor, “heightened emotion,” there were six words or phrases, such as “restless,” “strong,” and “leaping” (Cronbach’s α = 0.789).

**Table 2:** Findings of the factor analysis on emotional response to music.

	Factor 1	Factor 2	Factor 3
**Emotion-related items**			
Tender	**0.931**	0.007	-0.209
Fascinated	**0.927**	0.055	-0.169
Peaceful	**0.923**	0.051	-0.279
Serene	**0.911**	0.026	-0.204
Easy	**0.885**	0.133	-0.243
Relaxed	**0.874**	0.069	-0.135
Tranquil	**0.851**	0.035	-0.019
Warm	**0.814**	-0.089	0.062
Heartwarming	**0.803**	-0.060	0.051
Dreamy	**0.774**	0.030	0.105
Familiar	**0.769**	0.206	-0.027
Light	**0.757**	-0.079	0.056
Nostalgic	**0.712**	0.068	-0.128
Refreshing	**0.696**	-0.194	0.089
Happy	**0.691**	-0.173	0.176
Generous	**0.690**	0.209	0.022
Soothing	**0.677**	0.239	-0.007
Gorgeous	**0.651**	-0.069	0.041
Shining	**0.651**	-0.023	0.266
Animated	**0.648**	0.116	0.220
Joyful	**0.630**	-0.211	0.242
Amused	**0.590**	-0.205	0.285
Cheerful	**0.585**	-0.254	0.305
Blithe	**0.550**	-0.035	0.278
Yearning	**0.527**	0.158	0.183
Feel like dancing	**0.519**	0.038	0.151
Lively	**0.465**	-0.236	0.400
Sad	0.082	**0.834**	-0.202
Lonely	0.205	**0.799**	-0.178
Weird	-0.144	**0.711**	0.348
Marbled	0.048	**0.709**	0.165
Gloomy	0.025	**0.707**	0.089
Fearful	-0.225	**0.690**	0.269
Disconsolate	-0.207	**0.689**	0.021
Heavy	-0.144	**0.659**	0.136
Weak	0.081	**0.634**	-0.043
Horrible	-0.152	**0.607**	0.333
Restless	-0.235	0.311	**0.657**
Stately	-0.022	-0.038	**0.628**
Strong	0.194	0.115	**0.557**
Leaping	0.058	0.426	**0.459**
Lofty	0.276	0.216	**0.407**
Filled with Wonder	0.049	0.308	**0.401**
**Correlations between factors**			
Factor 1	-	-0.219	0.361
Factor 2		-	-0.004
Factor 3			-

*Bolded values indicate items that loaded on the factor represented in that column.*

We then conducted a path analysis to evaluate the model proposed in this study, and to test the hypothesis that the sub-components of trait empathy relate to preference for sad music through emotional response.

### Model Testing

Path analyses were performed to test the proposed model hypothesized that the sub-components of trait empathy have a direct and indirect association with liking sad music, and that emotional responses to music mediate the indirect association.

**FIGURE 2:**
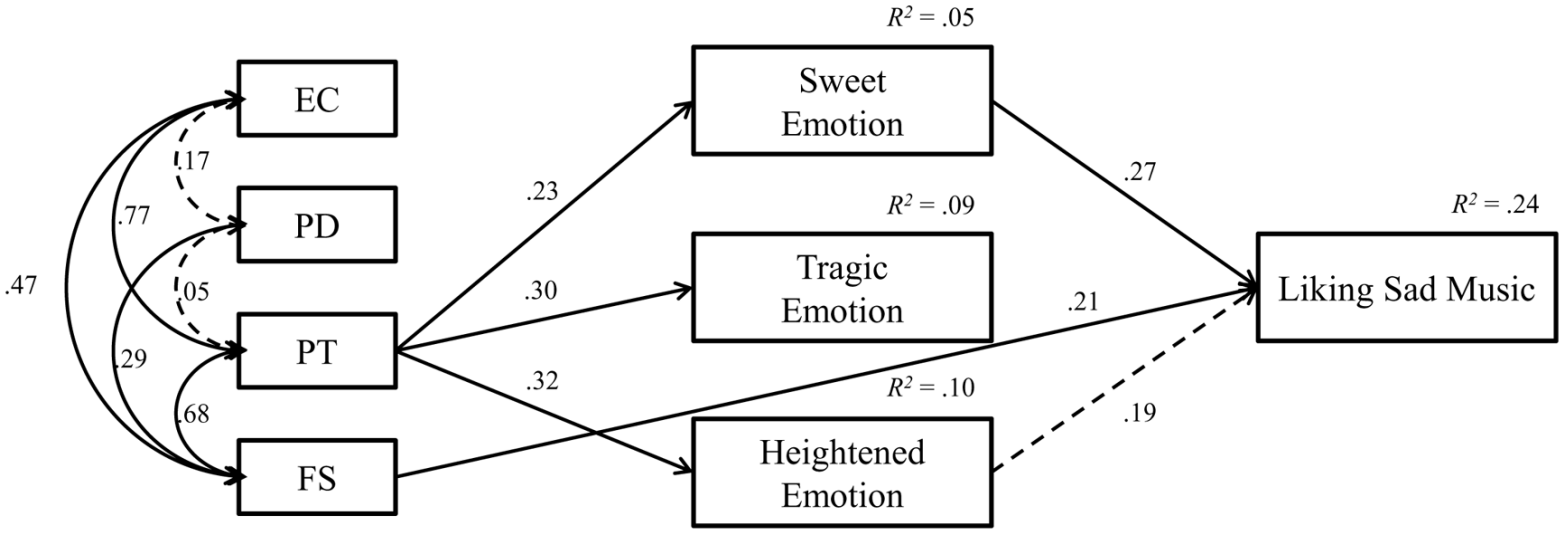
**Path analysis of the modified model**.

**Figure 2** shows the final model including the standardized regression weights and *R*^2^ values. Dashed lines indicate non-significant associations. The resulting fit indices for the path analysis were as follows: the chi-square value was not significant, indicating that the model was consistent with the data; and the AGFI (0.928), GFI (0.974), and RMSEA (0.000) values were acceptable.

The results of the path analysis indicated that some sub-components of trait empathy are directly related to the preference for sad music, while the others were associated with emotional responses to music stimuli, and were then associated with preference for sad music. Among the sub-components of trait empathy, only FS showed a significant direct association with liking sad music (the standardized regression weight was 0.21). Only PT demonstrated a significant association with emotional response, in that it was positively associated with all the three musical emotion factors: sweet, tragic, and heightened emotions (the standardized regression weights were 0.23, 0.30, and0.32, respectively). Furthermore, sweet emotion was positively associated with liking sad music (the standardized regression weight was 0.27). While non-significant, the positive relationship of liking sad music and heightened emotion contributed to the variance explained by the model.

## Discussion

### Relationships between the Sub-components of Trait Empathy and Liking Sad Music

Previous studies have suggested that trait empathy was associated with liking sad music. For example, Garrido and Schubert (2011) showed that there was a correlation between EC and liking sad music. Vuoskoski et al. (2012) suggested that the degree of liking sad music was correlated with EC, FS, and global empathy. In line with these studies, we found that liking sad music was significantly correlated with both EC [*r*(84) = 0.28, *p* < 0.05] and FS [*r*(84) = 0.27, *p* < 0.05]. In addition, the current data demonstrated that PT was significantly correlated with liking sad music [*r*(84) = 0.35, *p* < 0.01], while previous studies did not find this association.

This difference in the present results as compared from those from previous studies (Garrido and Schubert, 2011; Vuoskoski et al., 2012) could be explained in a few ways. First, Garrido and Schubert (2011) used only the EC and FS scales of the IRI. They did not use PT scale, and therefore, did not find a relationship with PT. Second, the age of the participants might have affected the results. Vuoskoski et al. (2012) used four sub-scales of the IRI as in the current study, but their results did not show a relationship between PT and liking sad music. However, the participants in the studies reported by both Garrido and Schubert (2011) and Vuoskoski et al. (2012) were adults, while those in the current study were elementary school children. This might account for the difference in our results.

Furthermore, nationality might influence the results of this investigation. Wu and Keysar (2007) recorded eye-gaze and found that Chinese participants were more in tune with their partner’s perspective than were American participants. If perspective-taking ability varies widely across cultures, the components of trait empathy that correlates with liking sad music would also differ.

### A Model of Trait Empathy, Liking Sad Music, and Emotional Response to Music

**FIGURE 3:**
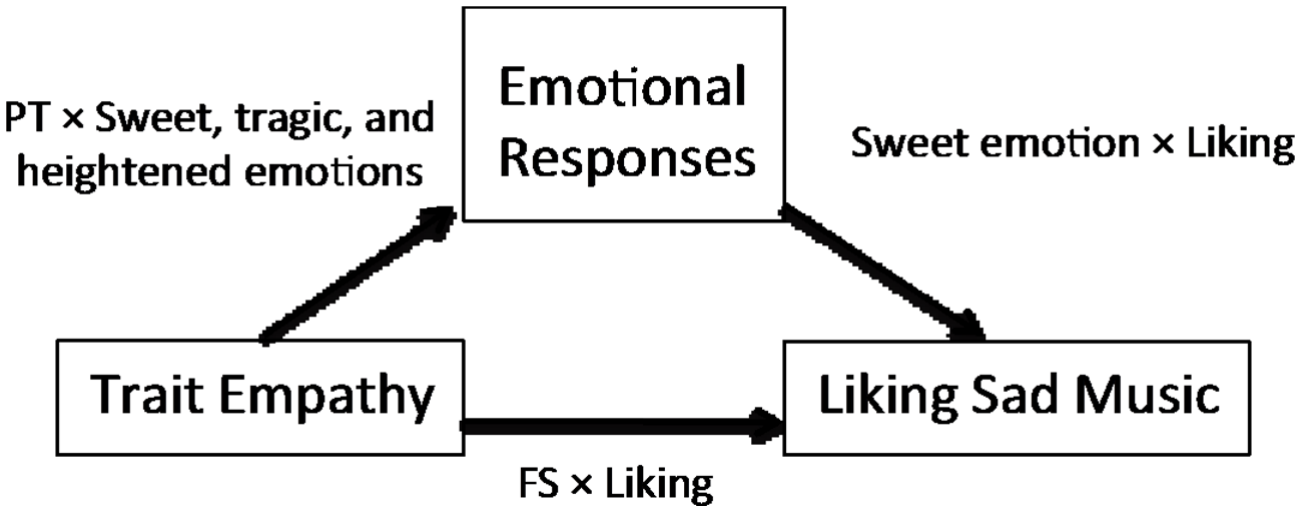
**The model proposed in the current study**.

In the current work, we performed a path analysis of a modified model and found that trait empathy was associated with preference for sad music both directly and indirectly. According to us, this indicates that FS affected the preference for sad music directly and PT affected it indirectly, mediated by one’s emotional response to the music (**Figure 3**). Our findings demonstrate that children with a high perspective-taking ability tended to experience ambivalent emotions such as sweet and tragic emotions, as well as heightened emotions. In addition, pleasant emotion like sweet emotion was particularly associated with a preference for sad music (**Figure 3**).

Others have considered the pairwise relationships between trait empathy and liking sad music or that between trait empathy and an emotional response to music. However, our results offer a more elaborate characterization of the relationships among trait empathy and emotional response to and preference for sad music (**Figure 3**). Previous studies have reported that liking sad music is associated with EC (Garrido and Schubert, 2011; Vuoskoski et al., 2012) and FS (Vuoskoski et al., 2012). The path analysis results in the present study suggested a direct influence of FS on liking sad music, while we did not find a direct or indirect influence of EC. We suspect that this is partly explained by the correlation between EC and FS. In the current study, EC was correlated with FS (*r* = 0.47, *p* < 0.01). Perhaps the effect of EC reported in earlier studies was mediated by FS. However, our results can be interpreted as supporting the idea that FS has an effect on liking sad music.

Based on our results, we can say that FS affects preference for sad music irrespective of one’s emotional response to music. According to Mar et al. (2006), readers with high FS scores prefer fictional work. In addition, higher scores on the Openness to Experience dimension are related to increased esthetic sensitivity, and FS correlates with Openness to Experience (Mar et al., 2009). Taken together, these findings suggest that FS ability is relevant for positive reactions toward imaginative art, including music.

Is Self-other Differentiation the Key to Experience Pleasant Emotions in Response to Sad Music?

One finding specific to the current study was that PT associates with preference for sad music via one’s emotional response to sad music. Meanwhile, it has been pointed out that the general strength of one’s emotional response to music correlates with liking sad music (Ladinig and Schellenberg, 2012; Vuoskoski et al., 2012), the present study demonstrated that positive emotion, such as sweet emotion, was related to liking sad music. If people experience only sadness while listening to sad music, they are less likely to prefer it. Our results suggested that people like sad music if they experience pleasant emotions in response to hearing it.

The association of PT with emotional response to sad music is a new finding obtained in the present study. According to Eisenberg et al. (1991), when participants watched a film that induced sympathy and focused on emotional content, PT showed a positive correlation with vicarious emotional reactions. If PT is a prerequisite or promotional factor for vicarious emotions, emotional responses in this study were also strengthened by perspective-taking ability. In addition, it is interesting that PT was found to be associated with a positive emotional response to sad music. Lamm et al. (2008) noted that the self-other differentiation is associated with PT. We speculate that while sad music exerts a negative emotional influence (sadness) on listeners, the ability for self-other differentiation might enable them to assess the emotional stimulus objectively and leave room for the experience of more positive emotions. Such interpretation seems to be congruent with a notion put forth by Kawakami et al. (2013b) that emotional responses to sad music could bear positive properties because there is no real threat. A similar idea is found in the concept of “sublime” that was proposed by Kant (2000) to describe an esthetic experience of objects which induce fear in a perceiver. In order for a pleasurable experience to arise by perceiving stimuli that have negative emotional qualities, self-other differentiation, and the awareness of the absence of threat that such a differentiation allows, might serve a crucial role.

On the other hand, in the current study FS did not have a significant effect on emotional response to the sad music, despite the report by Vuoskoski et al. (2012) that the intensity of emotions experienced in response to sad excerpts correlate with FS (*r* = 0.19, *p* < 0.05). As mentioned above, the strong effect of PT on emotional response to sad music in the present study might depend on the age of participants.

Considering that while self-centered pain appears in an earlier developmental phase, perspective taking and other-centered empathy emerge in later childhood (Hoffman, 2000). Indeed, later in their development, children might tend to experience more pleasant emotions when listening to sad music and therefore prefer it. Hasegawa et al. (2009) demonstrated that fourth- and fifth-grade elementary school students are at a point of demarcation between self-centered pain and perspective-taking. It is possible that PT strongly influenced the emotional response to sad music in this study because there were large developmental differences in PT across children. To verify this possibility, we will need to examine this relationship in adult participants.

### Limitations of the Current Study

In the current study, we examined the relationships between trait empathy and responses to sad music in a sample of sixth-grade children. The relationship between interpersonal empathy and emotional response to music is in itself an interesting issue. However, further studies including other aspects of interpersonal empathy are needed to elucidate the underlying mechanism. The current study offers useful insights toward this end. However, several limitations of the present study must be discussed.

First, the present study used only sad music as experimental stimuli. It should be carefully assessed whether the relationships between trait empathy and responses to sad music shown in this study were unique to sad music. In the previous studies, openness to experience, which was reported to be associated with FS, was also reported to be related with the preference for sad music exclusively (Ladinig and Schellenberg, 2012; Vuoskoski et al., 2012). Additionally, FS was reported to be related with a preference for not only sad music, but also for tender music (Vuoskoski et al., 2012). It is therefore necessary to examine the relationships between the sub-components of trait empathy and music preferences related to emotions other than sadness.

Secondly, the developmental aspects of empathic ability might have influenced the results because only elementary school children participated in the present study. At first, we assumed that this highlights the individual differences in empathy, and thereby contributes to the goal of this study. However, only a part of the sub-components of trait empathy, which were obtained at the age group of the participants in this study, might be enhanced. Moreover, a previous study (Ladinig and Schellenberg, 2012) reported that music training positively correlated with liking toward a type of music that evokes mixed emotions (co-occurrence of positive and negative emotions). Although negative emotions did not relate to liking for sad music in the present study, positive and negative emotions may show different tendencies in adults or adolescents due to their accumulated musical experience.

For the reasons mentioned above, future work should focus on a wider age range and examine whether the personal traits that determine one’s emotional response to sad music differ according to the phase of development. We are currently designing a study to address these issues.

Finally, the sample size was small in the present study (*n* = 84), which means that the results obtained in this study must be carefully interpreted and should be verified in a future study with a larger sample size.

## Conclusion

In sum, our results indicated that, of the sub-components of trait empathy, perspective-taking ability affected the emotional response to sad music (e.g., sweet, tragic, and heightened emotions). In addition, the data suggested that sweet emotions contributed to the preference for sad music, while the tendency to fantasize explained the preference for sad music directly. The results of this study demonstrate how human empathy associates with the emotional response to music in a comprehensive manner.

## Conflict of Interest Statement

The authors declare that the research was conducted in the absence of any commercial or financial relationships that could be construed as a potential conflict of interest.
